# Cryotherapy shows no inferiority compared with radical Prostatectomy for low-risk and intermediate-risk localized Prostate Cancer: a real-world study from the SEER database

**DOI:** 10.7150/jca.38323

**Published:** 2020-07-29

**Authors:** Kun Jin, Shi Qiu, Xiaonan Zheng, Yanyan Li, Shiyu Zhang, Jiakun Li, Xinyang Liao, Xiang Tu, Lu Yang, Qiang Wei

**Affiliations:** 1 Department of Urology, Institute of Urology and National Clinical Research Center for Geriatrics, West China Hospital, Sichuan University, No. 37 Guoxue Xiang, Chengdu 610041, China.; 2 Center of Biomedical big data, West China Hospital, Sichuan University, Chengdu, Sichuan, China.

**Keywords:** Cryotherapy, Radical Prostatectomy, Localized, Prostate Cancer

## Abstract

**Background:** For localized prostate cancer (PCa) with a low disease burden, whole-gland resection seems like overtreatment, while focal therapy, including cryosurgery, can achieve similar outcomes. We aimed at comparing the long-term survival outcomes of cryotherapy and radical prostatectomy (RP) and further exploring whether RP can be replaced by cryosurgery for those with low-risk PCa.

**Methods:** We conducted analyses from the Surveillance, Epidemiology, and End Results (SEER) database (2004-2015) and performed propensity score matching and used an instrumental variate to reduce the influence of bias and unmeasured confounders to the greatest extent.

**Results:** In the multivariate regression, patients who received cryotherapy had higher risk of overall mortality (OM) (hazard ratio [HR] = 2.52, 95% confidence interval [CI] 1.99-3.20, p < 0.001), but no significant difference was observed in decreasing cancer-specific mortality (CSM) (HR = 1.38, 95% CI 0.63-3.03, p = 0.41) after adjusting the confounders. After propensity score matching, patients who underwent cryotherapy had higher OM and CSM rates (HR = 2.70 [95% CI 1.99-3.66, p < 0.001] and HR = 2.99 [95% CI 1.19-7.48, p = 0.02], respectively). In the IV-adjusted analyses, RP was superior to cryotherapy in decreasing OM (HR = 2.52, 95% CI 1.99-3.20), while no obvious decrease of CSM was observed in the comparison of RP and cryotherapy (HR = 1.38, 95% CI 0.63-3.03). The subgroup analyses showed that RP displayed an obvious benefit in decreasing CSM (HR = 5.02, 95% CI 1.30-19.39, p = 0.02) for those with a prostate-specific antigen (PSA) level higher than 10 ng/ml.

**Conclusion:** RP ranked as the best treatment in regard to tumor control, but the advantages of cryotherapy became evident when taking functional and oncological outcomes into account, especially for low- and intermediate-risk PCa with low PSA levels.

## Introduction

Prostate cancer (PCa), the third most common malignancy in the USA, is ranked the sixth leading cause of cancer death in males, with an estimated 160,000 new cases diagnosed in 2017 1,2. With the widespread use of prostate-specific antigen (PSA) testing, an increasing number of men are diagnosed with localized PCa with a lower clinical stage, smaller volume and lower grade. Active surveillance is suggested for most individuals, due to the slight effect on overall survival. Once tumor progression occurs, radical treatments including radical prostatectomy (RP) and radiation therapy are suitable options according to EAU guidelines 3. Although these interventions have long-term oncological control outcomes, side effects on genitourinary function cannot be avoided, such as incontinence, urinary symptoms, erectile function, and rectal side effects 4,5. For those with low-grade and small-size PCa, whole-gland resection seems like overtreatment, while focal therapy, including cryosurgery, can limit toxicity by sparing the neurovascular bundles, sphincter, and urethra 6-8.

According to one single-arm research study including 370 individuals who underwent cryosurgery, results showed that the 10-year biochemical disease-free survival rates were 80.6% and 74.2% for the low- and intermediate-risk groups 9. However, direct comparative data and long-term oncological outcomes are still lacking, as the relevant studies focus mainly on non-comparative single-arm case series 10. As a result, no consensus has been reached on whether cryosurgery could be an alternative therapy to cure localized PCa.

Our study aims at comparing the long-term survival outcomes between cryotherapy and radical prostatectomy and further exploring whether RP can be replaced by cryosurgery for those with low-risk PCa.

## Methods

### Patient selection

**Figure 1:**
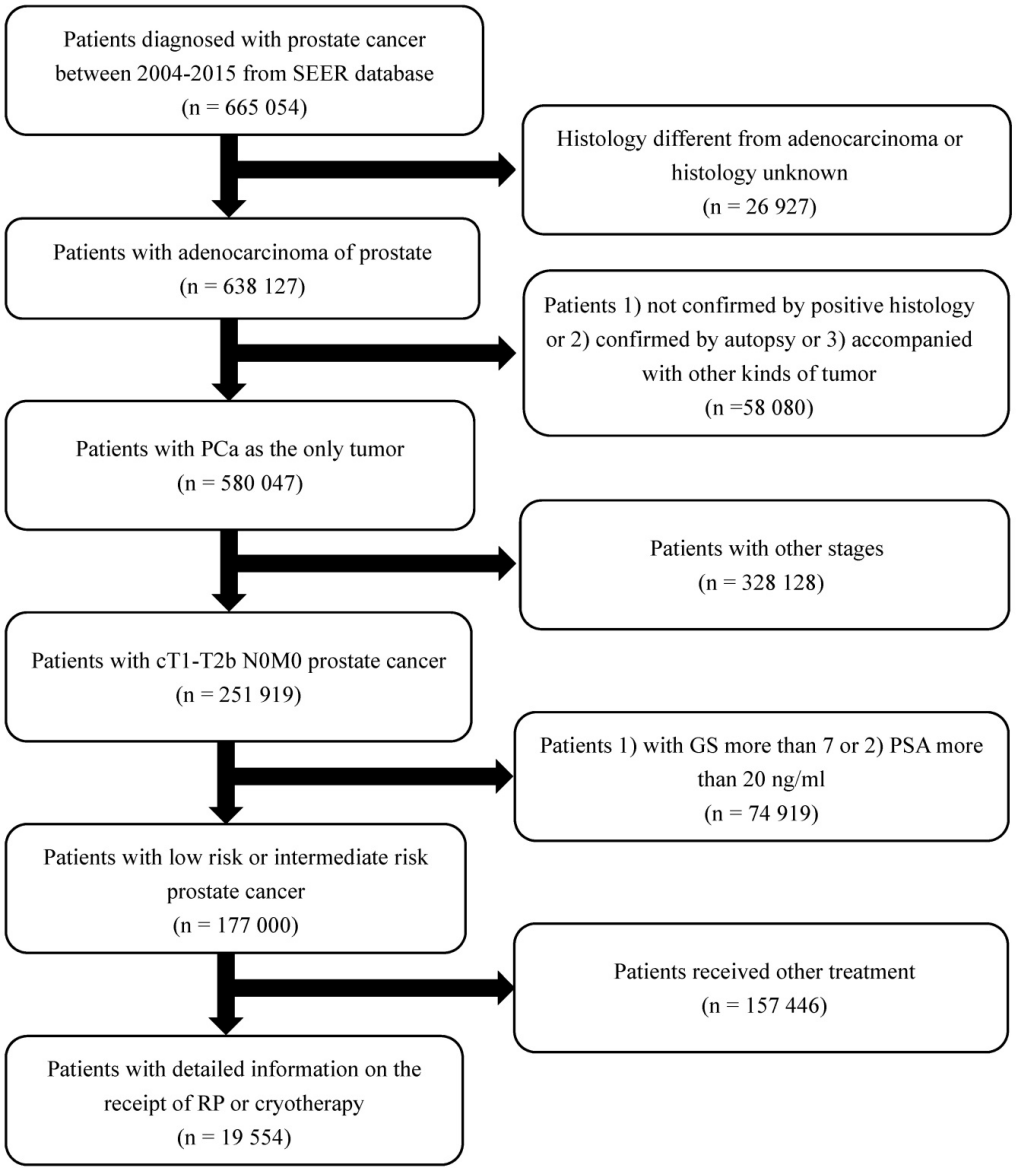
Flowchart describing the selection of patients treated with local treatment or non-local treatment in the Surveillance, Epidemiology and End Results databases, 2004-2015. Abbreviation: RP: radical prostatectomy.

From the Surveillance, Epidemiology, and End Results (SEER) database, consisting of 18 cancer registries in America and accounting for 26% of the US population, we identified patients diagnosed with adenocarcinoma of the prostate (International Classification of Diseases-O-3 code: C61.9) between 2004 and 2015. The TNM stages were evaluated according to the sixth edition of the American Joint Committee on Cancer Staging Manual 11. Inclusion and exclusion criteria are shown in detail in the flowchart (Figure 1). Patients were stratified into two treatment groups: cryotherapy and RP. These selection criteria yielded 19,554 patients in total.

### Propensity score matching

To emulate randomized cohort trial design and minimize selection bias, we performed propensity score matching (1:1 ratio, with nearest-neighbor matching or caliper width of 0.05), which yielded similar patient characteristics between cryotherapy (n = 2,350) and RP cohorts (n = 17,204). Adjustment variables consisted of age, biopsy Gleason score (GS), clinical tumor stage, and PSA level.

### Statistical analysis

First of all, in the analysis of baseline characteristics, differences in continuous variables were evaluated with a two-tailed *t* test and presented as the mean ± standard deviation, whereas differences in categorical variables were compared with a two-tailed χ^2^ test (or Fisher's exact test) and presented as frequency with its proportion. Second, Cox proportional hazards regressions were performed to assess cancer-specific mortality (CSM) and overall mortality (OM) between treatment groups in the crude models and adjusted-covariate models. In the process of matching, propensity scores were estimated with logistic regression, with treatment (cryotherapy and RP) as the outcome and age, clinical T stage, GS, and PSA level as pretreatment, prognostic covariates. The matched baseline characteristics between the two groups were regarded as balanced when *p* > 0.05. In the original cohort, cumulative incidence survival curves were obtained with Kaplan-Meier methods.

Considering the selection bias and unmeasured confounders, we additionally used an instrument variate (IVA) to calculate them. We selected the regional utilization rate as the IVA in the two-stage residual inclusion analysis 12-15. Afterward, another multivariate Cox proportional hazard model, including all covariates and residuals, was presented to illustrate the results more precisely.

Several sensitivity analyses were performed to validate the robustness of the results: (1) analyses of CSM and OM after adjusting propensity scores; (2) inverse probability of treatment weighing (IPTW) and standardized mortality ratio weighting (SMRW) calculated with the propensity score to estimate the relationship between treatment types and outcomes among the entire cohort; (3) analyses of CSM and OM stratified by propensity scores; and (4) adjustment of unbalanced covariates in the matched cohort.

All analyses were performed with the statistical software packages R (http://www.R-project.org, The R Foundation) and EmpowerStats (http://www.empowerstats.com, X&Y Solutions, Inc., Boston, MA). A *p* value of <0.05 was considered statistically significant.

## Results

Our study included 19,554 individuals, of who 2,350 received cryotherapy, while 17,204 received RP. The inclusion and exclusion criteria are presented in detail in Figure 1. Table 1 shows the patients' baseline characteristics. Patients who received cryotherapy had a higher average age and PSA level than those who received RP. However, the T stage and GS were lower in the cryotherapy group. From the multivariate regression analysis, patients who received cryotherapy had a higher overall risk of death (hazard ratio [HR] = 4.74, 95% confidence interval [CI] 4.20-5.35, *p* < 0.001). After adjusting the covariates, including age, PSA, T stage, and GS, cryotherapy still led to a higher overall risk of death than RP (HR = 2.52, 95% CI 1.99-3.20, *p* < 0.001). As for the CSM analyses, results from the crude model indicated that cryotherapy was inferior to RP in decreasing CSM (HR = 4.81, 95% CI 3.26-7.09, *p* < 0.001). However, no significant differences were observed after adjusting confounders, with HR = 1.38 (95% CI 0.63-3.03, *p* = 0.41).

**Figure 2:**
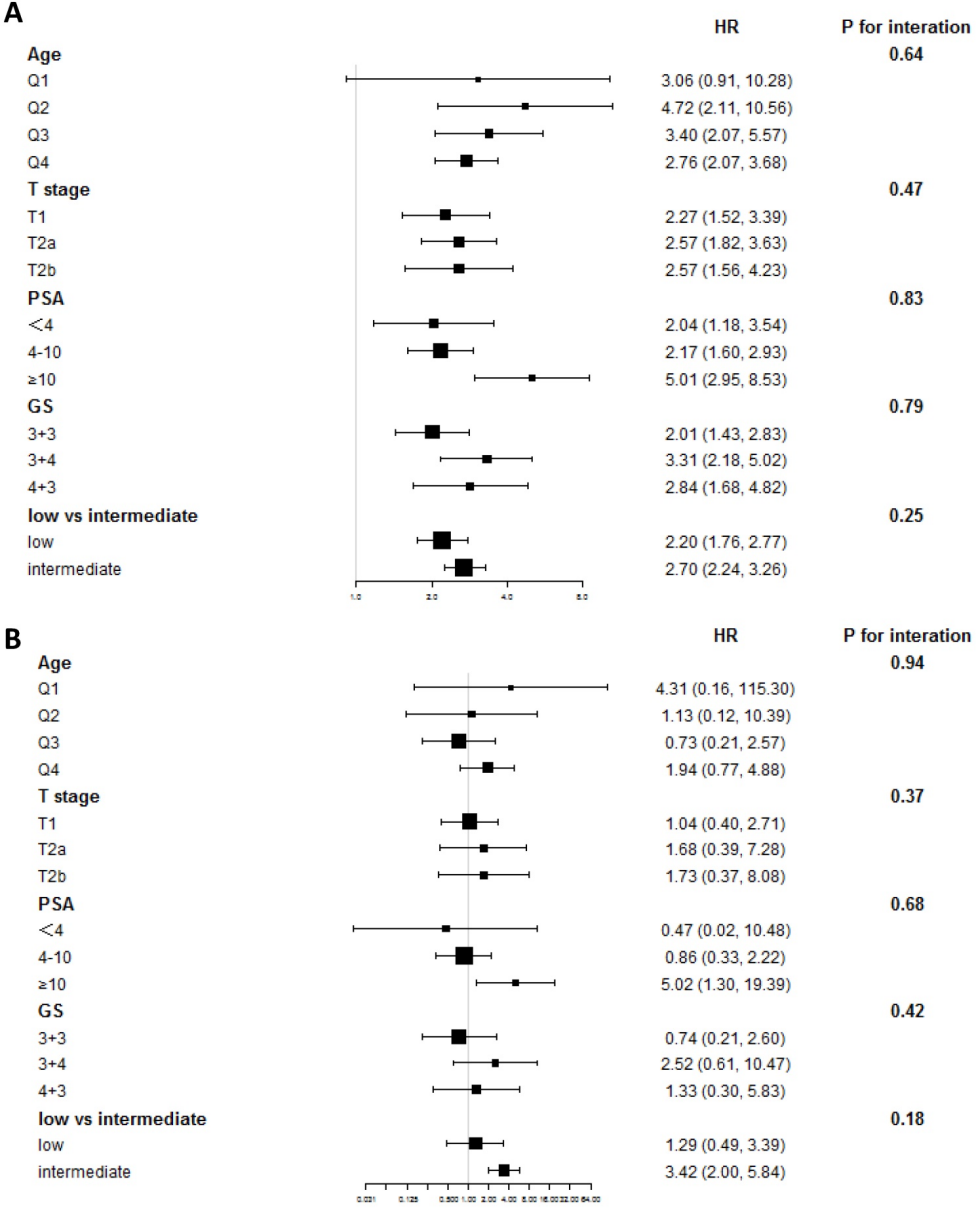
** Subgroup analyses of OM and CSM in the comparison between RP and cryotherapy.** (**A**) Subgroup analysis of OM in the comparison of RP and cryotherapy. (**B**) Subgroup analysis of CSM in the comparison of RP and cryotherapy. Abbreviations: OM: Overall mortality; CSM: Cancer specific mortality; RP: Radical prostatectomy; GS: Gleason Score; PSA: Prostate specific antigen; Q1-Q4: Quartile 1 - Quartile 4.

After propensity score matching, a total of 2,060 patients were screened in the matched cohort, with 1,030 in each treatment group. After matching, the age, T stage, and GS remained unbalanced (Table 2). In the matched cohort, patients who had undergone cryotherapy had higher OM and CSM (HR = 2.70 [95% CI 1.99-3.66, *p* < 0.001] and HR = 2.99 [95% CI 1.19-7.48, *p* = 0.02], respectively). After adjusting the imbalanced covariates in the matched cohort, patients who had undergone RP showed a higher survival rate than those who had undergone cryotherapy (HR = 2.19, 95% CI 1.60-3.00, *p* < 0.001), while cryotherapy showed non-inferiority in the aspect of CSM (HR = 1.76, 95% CI 0.68-4.52, *p* = 0.24). In the IV-adjusted analysis (Table 3), cryotherapy was inferior to RP in decreasing OM (HR = 2.52, 95% CI 1.99-3.20), while no obvious decrease of CSM was observed in the comparison of RP and cryotherapy (HR = 1.38, 95% CI 0.63-3.03). The subgroup analyses showed that patients with different PSA levels had different results from RP and cryotherapy. Stratified by the PSA level, RP displayed obvious benefits in decreasing CSM (HR = 5.02, 95% CI 1.30-19.39, *p* = 0.02) (Figure 2). We further used the D'Amico risk classification to divide the patients into low- and intermediate-risk groups. The same benefit of RP was found among those with intermediate-risk PCa (HR = 3.42, 95% CI 2.00-5.84, *p* < 0.001), while no significant outcome was observed in GS = 7 [(HR = 2.52, 95% CI 0.61-10.47, *p* = 0.20) for GS = 3+4 and (HR = 1.33, 95% CI 0.30-5.83, *p* = 0.70) for GS = 4 + 3], indicating that the PSA level played a more important role than GS.

The sensitivity analyses demonstrated similar survival outcomes in the comparison of cryotherapy and RP. The IPTW- and SMRW-adjusted models both showed no clear superiority in terms of reducing CSM after the adjustment of confounders (HR = 0.88, 95% CI 0.67-1.15, *p* = 0.35 and HR = 0.67, 95% CI 0.41-1.10, *p* = 0.12, respectively) (Table S2). The propensity score-adjusted model also reached the same outcome. With regard to a decline in CSM, no obvious differences were observed between the two therapies.

## Discussion

Currently, cryotherapy occurs as a new primary or salvage treatment of PCa since its first use 16,17. Cryotherapy induces tumor apoptosis by promoting protein denaturation and the destruction of cellular membranes by using extremely low temperature, which then leads to vascular stasis and microthrombi and causes ischemic necrosis of the tumor tissue 18-21.

Several studies show that focal cryotherapy presents encouraging short-term outcomes 22-25. However, most of these studies were single-arm designed, with few focusing on the effectiveness comparison of RP and cryotherapy. Our study examined long-term oncologic outcomes with a follow-up duration of 72 months (range: 43-103 months). According to our results, for low- and intermediate-risk PCa, cryotherapy shows no significant difference in decreasing CSM compared with RP, but is also no match for RP in OM decline.

Additionally, one retrospective matched-pair cohort including 68 individuals in each group showed that oncologic outcomes after focal cryosurgery versus RP were similar in regard to the need for salvage therapy (*p* = 0.55) 26. This research also reported that those who received cryosurgery had a 70% reduction in the PSA level (pre-cryotherapy means PSA 5.9 ng/ml and post-cryotherapy means PSA 1.6 ng/ml), indicating that cryosurgery indeed limited the progression of the disease, with an effectiveness similar to RP. In a larger sample size study (n = 1160), patients who received focal cryotherapy showed a 75.7% biochemical recurrence-free rate at 36 months 27, and the postoperative biopsy was positive in only 3.7% of patients. Nevertheless, survival outcomes after cryotherapy were not analyzed in this study. Another single-arm study, including 26 participants who underwent focal cryosurgery of the prostate, reported that only three cases experienced biochemical failure, and after salvage conventional definitive therapy, they recovered favorable PSA nadirs 28. Similar function preservations of cryotherapy were observed in other studies 29-31, but all these failed to compare cryotherapy and RP in the analysis of long-term survival outcomes.

Most research has focused on low- and intermediate-risk PCa, while several studies included patients of high risk. According to Masakatsu Oishi et al. 32, among those who underwent cryotherapy, high-risk patients were more likely to suffer clinical recurrence (*p* = 0.046), based on the Kaplan-Meier survival curve and trend toward statistical significance (*p* = 0.06) on multivariate analysis. Therefore, the authors suspected that cryotherapy might not be suitable for high-risk PCa, but could become an alternative option for low- and intermediate-risk PCa. Another prospective study that enrolled intermediate- and high-risk individuals showed no discrepancy between the two risk groups 33. More interestingly, when stratified based on PSA levels at 10 ng/ml, subgroup analyses showed that patients with PSAs higher than 10 ng/ml were more likely to develop disease recurrence. In this study, the PSA level played a more important role than GS for high-risk PCa, while similar results were observed in our study for those with intermediate-risk PCa. However, more research is needed to explore whether high-risk participants with low PSA levels could benefit from cryotherapy in regard to oncological outcomes, with the exception of functional outcomes.

A major strength of our data comes from the large sample database and direct comparison of cryotherapy and RP. We performed various analyses to verify the effect of cryotherapy on tumor control. The results all showed that cryotherapy was similar to RP in decreasing CSM. Most studies have focused on the short-term outcomes, such as biochemical recurrence and clinical failure. Our research further explored the survival outcomes and could prove to be a valuable reference in choosing the proper treatment.

Some limitations also need to be highlighted. First, the nature of the retrospective study could not be overcome entirely, although we performed propensity score matching to assume randomization. Second, other assessments of patients were not recorded in the SEER database, such as the Charlson Comorbidity Index and the ECOG score. However, we used an IVA to calculate the unmeasured confounding and make the result more precise. Third, functional outcomes, including urinary incontinence and ejaculation dysfunction (ED), were also not presented in the database. According to the present studies, 0%-3.6% incontinence and 0%-42% ED rates were reported among those who received cryotherapy 34, with fewer complications than RP. In addition, outcomes including disease recurrence and biochemical recurrence were not assessed in our study due to the lack of relevant data. Additionally, as cryotherapy was not implemented in our center, we failed to perform the external validation by using data from our center, thus weaken the accuracy of our results.

## Conclusion

For those with low- and intermediate-risk PCa, cryotherapy could achieve results similar to RP in regard to tumor control. With regard to the preservation of functions, cryotherapy could become a better option. For patients with higher PSA levels, cryotherapy failed to provide survival benefits compared with those who underwent RP. In a word, RP ranked the best treatment in terms of tumor control, but the advantages of cryotherapy became evident when taking both functional and oncological outcomes into account, especially for low- and intermediate-risk PCa with lower PSA levels.

## Supplementary Material

Supplementary figures and tables.

## Figures and Tables

**Figure 3:**
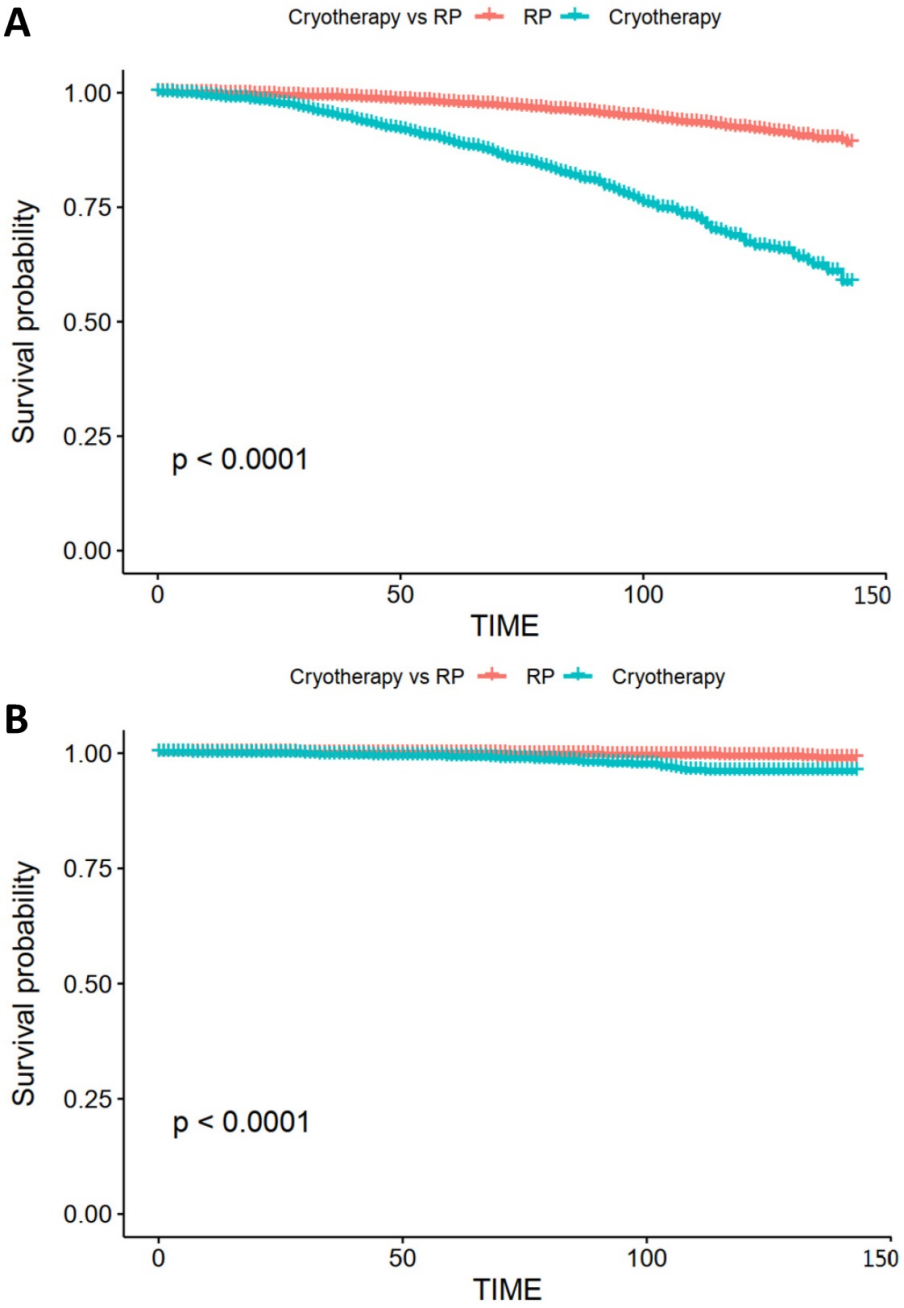
** Kaplan-Meier survival curve of OS and CSS.** (**A**) Kaplan-Meier survival curve of OS in the comparison of RP and cryotherapy. (**B**) Kaplan-Meier survival curve of CSS in the comparison of RP and cryotherapy. Abbreviations: OS: Overall survival; CSS: Cancer specific survival; RP: Radical prostatectomy.

**Table 1:** Descriptive characteristics of 19,554 patients that underwent either cryotherapy or radical prostatectomy between 2004 and 2015 from the Surveillance Epidemiology and End Results database

	RP (N = 17204)	Cryotherapy (N =2350)	*P* value
**Age, yr mean** ± **SD**	60.32 ± 7.13	68.91 ± 7.55	<0.001
**PSA level (ng/ml), mean** ± **SD**	5.85 ± 3.13	6.72 ± 3.35	<0.001
**Marital status, n (%)**			<0.001
Married	13460 (78.24%)	1658 (70.55%)	
Single	1596 (9.28%)	220 (9.36%)	
Divorced/Widowed	1204 (7.00%)	256 (10.89%)	
Unknown	944 (5.49%)	216 (9.19%)	
**Race, n (%)**			<0.001
White	14522 (84.41%)	1857 (79.02%)	
Black	1518 (8.82%)	370 (15.74%)	
Other	981 (5.70%)	97 (4.13%)	
Unknown	183 (1.06%)	26 (1.11%)	
**T stage, n (%)**			<0.001
T1	720 (4.19%)	1993 (84.81%)	
T2a	13827 (80.37%)	242 (10.30%)	
T2b	2657 (15.44%)	115 (4.89%)	
**Gleason Score, n (%)**			<0.001
3+3	10406 (60.49%)	1135 (48.30%)	
3+4	5178 (30.10%)	827 (35.19%)	
4+3	1620 (9.42%)	388 (16.51%)	
**Low risk vs intermediate risk, n (%)**			<0.001
Low risk	8388 (48.76%)	967 (41.15%)	
Intermediate risk	8816 (51.24%)	1383 (58.85%)	

Abbreviations: SD: standard difference; PSA: prostate-specific antigen.

**Table 2:** Descriptive characteristics of 2,060 patients received RP versus cryotherapy after propensity score matching (ratio 1:1) between 2004 and 2015 from the Surveillance Epidemiology and End Results database

	RP	Cryotherapy	P value
**Age, yr mean ± SD**	63.70 ± 7.78	65.03 ± 8.06	<0.001
**PSA level (ng/ml), mean** ± **SD**	6.44 ± 3.43	6.50 ± 3.73	0.71
**Marital status**			<0.001
Married	787 (76.4)	705 (68.4)	
Single	78 (7.6)	116 (11.3)	
Divorced/Widowed	77 (7.5)	119 (11.6)	
Unknown	88 (8.5)	90 (8.7)	
**Race**			<0.001
White	873 (84.8)	804 (78.1)	
Black	87 (8.4)	178 (17.3)	
Other	58 (5.6)	36 (3.5)	
Unknown	12 (1.2)	12 (1.2)	
**T stage**			<0.001
T1	636 (61.7)	673 (65.3)	
T2a	340 (33)	242 (23.5)	
T2b	54 (5.2)	115 (11.2)	
**GS biopsy**			0.0071
3+3	578 (56.1)	510 (49.5)	
3+4	311 (30.2)	343 (33.3)	
4+3	141 (13.7)	177 (17.2)	
**Low risk vs intermediate risk**			0.0005
Low risk	474 (46)	395 (38.3)	
Intermediate risk	556 (54)	635 (61.7)	

Abbreviations: SD: standard difference; PSA: prostate-specific antigen; RP: radical prostatectomy.

**Table 3:** Multivariate cox regression analyses for CSM and OM in the total cohort and matched population

Outcome	Treatment	Non-adjusted model	Adjusted model	PSM model	IVA-adjusted model
CSM	RP	Ref.	Ref.	Ref.	Ref.
	cryotherapy	4.81 (3.26, 7.09) p<0.0001	1.38 (0.63, 3.03) p=0.4162	2.99 (1.19, 7.48) p=0.0195	1.38 (0.63, 3.03)
OM	RP	Ref.	Ref.	Ref.	Ref.
	cryotherapy	4.74 (4.20, 5.35) p<0.0001	2.52 (1.99, 3.20) p<0.0001	2.70 (1.99, 3.66) p<0.0001	2.52 (1.99, 3.20)

Abbreviations: OM: overall mortality; CSM: cancer specific mortality; RP: radical prostatectomy; PSM: propensity score matching, IVA = instrument variable; Adjusted model: adjusted for age, T stage, Gleason score (GS) and prostate specific antigen (PSA) level; Propensity score matching (PSM) model: matched according to age, T stage, GS and PSA; Instrument variate (IVA) adjusted model: adjusted for age, T stage, GS and PSA and residual.
