# Supplementary material for: Cryotherapy shows no inferiority compared with radical Prostatectomy for low-risk and intermediate-risk localized Prostate Cancer: a real-world study from the SEER database
Source: J Cancer. 2020 Jul 29;11(19):5738–45. doi: 10.7150/jca.38323 (PMC7477459; doi:10.7150/jca.38323)
Supplement: Supplementary file 1 — Supplementary figures and tables. [file jcav11p5738s1.pdf]

Table S1: Propensity score parameter list

<table>
<tr><td>the variables used in calculating the propensity matching</td><td colspan="2">Age, PSA, GS, T stage</td></tr>
<tr><td>Propensity scoring algorithm</td><td colspan="2">Logistic regression model</td></tr>
<tr><td>C-statistical</td><td colspan="2">0.9618</td></tr>
<tr><td>Matching method</td><td colspan="2">Greedy matching within specified caliper distances</td></tr>
<tr><td>Distance metric</td><td colspan="2">0.05</td></tr>
<tr><td>Matching ratio</td><td colspan="2">(Radiation prostatectomy) 1:1 (cryotherapy)</td></tr>
<tr><td>Use of replacement</td><td colspan="2">With replacement</td></tr>
<tr><td rowspan="2">Matching sample size</td><td>RP: 1030 cases</td><td rowspan="2">Total: 2060 cases</td></tr>
<tr><td>Cryotherapy: 1030 cases</td></tr>
</table>

Table S2: sensitivity analysis from propensity score matching (PSM)

Several models were used to examine robustness of the treatment effects in the comparison of RP and cryotherapy:

(1) Inverse probability of treatment weighting (IPTW) logistic regression model: calculated by PS

(2) Standard mortality ratio weighting (SMRW) logistic regression model: calculated by PS

(3) Covariate adjustment propensity score (CAPS) model: adjusted for PS

(4) Double propensity score (DPS) adjusted model: adjusted for confounders in the matched cohort

(5) Propensity score (PPS) stratified model

**RP vs cryotherapy**	**OM**	**CSM**
**IPTW model**		
Non-adjusted	**3.39 (3.16, 3.62)**	**1.40 (1.13, 1.72)**
Adjusted	**2.17 (2.01, 2.35)**	0.88 (0.67, 1.15)
**SMRW model**		
Non-adjusted	**1.43 (1.21, 1.68)**	0.98 (0.61, 1.57)
Adjusted	1.11 (0.94, 1.33)	0.67 (0.41, 1.10)
**CAPS model**		
Non-adjusted	**4.74 (4.20, 5.35)**	**4.81 (3.26, 7.09)**
Covariate PS as continuous	**2.62 (2.06, 3.34)**	1.67 (0.75, 3.72)
Covariate PS as categorical (divided into five groups)	**3.02 (2.49, 3.65)**	**3.12 (1.69, 5.76)**
**DPS model**	**2.19 (1.60, 3.00)**	1.76 (0.68, 4.52)
**PPS stratified model**		
**Q1**	**5.78 (3.38, 9.88)**	**6.84 (1.55, 30.20)**
**Q2**	**13.10 (6.98, 24.59)**	**11.34 (1.43, 89.73)**
**Q3**	**3.07 (1.14, 8.29)**	1.00 (1.00, 1.00)
**Q4**	**2.81 (1.44, 5.48)**	3.13 (0.42, 23.38)
**Q5**	**2.55 (2.08, 3.14)**	**2.62 (1.34, 5.12)**

OS = overall survival, CSM = cancer specific mortality, PS = propensity score

IPTW model and SMRW model: adjust for age, prostate specific antigen, Gleason score, T stage

CAPS model: adjust for propensity score

DPS model: adjusted for age, Gleason Score, T stage
